# Surface Reconstruction‐Free Stability Achieving Highly Anticorrosive Seawater Splitting

**DOI:** 10.1002/advs.202516499

**Published:** 2025-10-27

**Authors:** Yanita Devi, Ruspika Sundaresan, Tilahun Awoke Zegeye, Mariel G. Tecson, Guan‐Hong Guo, Wei‐Chun Lin, Yu‐Cheng Shao, Ching‐Wei Tung, Chun‐Hu Chen

**Affiliations:** ^1^ Department of Chemistry National Sun Yat‐sen University Kaohsiung 80424 Taiwan; ^2^ Green Hydrogen Research Center National Sun Yat‐sen University Kaohsiung 80424 Taiwan; ^3^ Center for Environmental Sustainability and Human Health Ming Chi University of Technology New Taipei City 24301 Taiwan; ^4^ Department of Photonics National Sun Yat‐sen University Kaohsiung 80424 Taiwan; ^5^ National Synchrotron Radiation Research Center Hsinchu 300092 Taiwan; ^6^ Department of Materials Engineering Ming Chi University of Technology New Taipei City 24301 Taiwan

**Keywords:** antireconstruction catalyst design, chloride corrosion resistance, multimetallic oxide electrocatalyst, operando X‐ray absorption spectroscopy, seawater electrolysis

## Abstract

Surface reconstruction, although often associated with enhanced activity through the lattice oxygen mechanism (LOM), can expose vulnerable sites that accelerate chloride‐induced corrosion. It is demonstrated that a silver and cerium co‐doped iron manganese oxide catalyst achieves high oxygen evolution reaction (OER) activity while maintaining a stable, reconstruction‐free surface under alkaline seawater conditions. Operando X‐ray absorption spectroscopy (XAS), complemented by the previous operando Raman analysis, reveals no detectable rearrangement of chemical environment or electronic structures across the OER‐relevant potential window. The catalyst achieves a low overpotential of 210 mV to reach 10 mA cm^−2^ in alkaline seawater, while maintaining 99.5% faradaic efficiency for oxygen evolution. By adopting the electrocatalyst into an anion exchange membrane (AEM) electrolyzer (5×5 cm^2^ of active area) for direct seawater electrolysis, the durability tests show a stable electrolysis current of 5000 mA for at least 250 h, while the blank electrolyzer fails in 1 h with significant corrosion. These findings strongly support the hypothesis that inhibiting surface reconstruction can effectively enhance seawater corrosion resistance without sacrificing catalytic performance.

## Graphical abstract

The complex metal oxides of AgCe‐FeMnOH can achieve an excellent oxygen evolution (OER) activity while maintaining its stability under alkaline seawater electrolysis. The surface reconstruction‐free behaviors lead to the durable seawater corrosion resistance without compromising the catalytic activities.

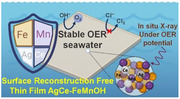

## 1 Introduction

Hydrogen production via seawater electrolysis is increasingly recognized as a promising pathway for sustainable energy systems.^[^ ^1^ ^]^ However, the highly corrosive nature of seawater—especially the abundance of chloride ions—poses serious challenges for long‐term catalyst stability.^[^ ^2^ ^]^ Despite significant advances in the development of oxygen evolution reaction (OER) electrocatalysts for alkaline water electrolysis, a critical knowledge gap remains unknown when translating these design principles to alkaline seawater environments.

The recent development of seawater OER catalysts focused on 1) repelling mechanisms to avoid Cl^−^ corrosion,^[^ ^3^, ^4^, ^5^, ^6^ ^]^ and 2) lattice stabilization to avoid active species leaching.^[^ ^7^ ^]^ For example, the OER catalysts incorporating barium ions (Ba^2+^) were reported to promote the adsorption of sulfate ions (SO_4_^2−^) and thus electrostatically repel chloride ions,^[^ ^8^ ^]^ but the adsorbed SO_4_^2−^ simultaneously can occupy the surface‐active sites and thus repelling the OH^−^ ions from performing OER, leading to a limited magnitude of current densities of water splitting. Other similar examples such as utilizing phosphate ions (PO_4_^3−^) have shown the similar problem.^[^ ^9^ ^]^ Besides the chloride repulsion strategy, robust electrocatalysts with negligible leaching is also essential for achieving corrosion resistance in seawater electrolysis. To address this, researchers have focused on minimizing structural reconstruction during electrolysis to improve catalyst durability.

Conventional wisdom in alkaline OER catalysis has long emphasized the necessity of surface reconstruction to achieve high activity.^[^ ^10^, ^11^, ^12^ ^]^ This restructuring, typically resulting in the formation of active oxyhydroxide layers,^[^ ^13^ ^]^ is viewed as essential step involving lattice oxygen mechanism (LOM) to enhance intrinsic activity.^[^ ^14^ ^]^ The participation of lattice‐oxygen on the surface could result in excessive oxygen vacancy that is irreversible and further leads to metal cation leaching^[^ ^15^, ^16^ ^]^ and structural instability.^[^ ^11^ ^]^ For example, in alkaline electrolyte, a CoFe*_x_* Al_2−_ *_x_* O_4_ spinel was reported to exhibit a primary leach of Al species, reconstructing the surface into a Co‐rich oxyhydroxide phase.^[^ ^17^, ^18^ ^]^ Although this dynamic leaching and reorganization can enhance activity by exposing reactive sites or forming phases more catalytically optimal than the original crystal structure, the drawback is that the water splitting performance cannot be maintained over multiple uses.^[^ ^19^ ^]^ We therefore hypothesize that designing electrocatalysts with suppressed surface reconstruction offers a promising strategy to significantly enhance corrosion resistance during alkaline seawater electrolysis.

Only a limited number of studies have reported catalyst systems that clearly recognize surface reconstruction‐free behavior during alkaline OER.^[^ ^20^ ^]^ More recently, Wu et al. recognized that suppressing surface reconstruction is essential for improving the stability of OER electrocatalysts in simulated seawater electrolysis,^[^ ^21^ ^]^ but a complete surface reconstruction‐free situation still has not been realized in their study. Thus, the direct correlation between the extent of durability of seawater electrolysis and surface reconstruction‐free (or not free) behaviors remains ambiguous and insufficiently understood.

Moreover, most of these studies rely predominantly on operando Raman spectroscopy to probe surface reconstruction.^[^ ^22^, ^23^ ^]^ While informative, this technique may overlook subtle or Raman‐inactive structural and chemical transformations. To gain a more complete understanding of reconstruction dynamics and their impact on catalytic performance, it is essential to incorporate complementary methods such as operando X‐ray absorption spectroscopy (XAS), which can directly probe whether the actual changes in oxidation states and local coordination environments occur or not.^[^ ^24^ ^]^

In our recent work, we identified an unusual behavior: high OER activity was achieved in complex oxides—specifically those composed of four different metal cation species—without invoking surface reconstruction.^[^ ^25^ ^]^ The operando Raman spectroscopy, Tafel slope analysis, and potential‐dependent activation energy measurements all confirmed the absence of surface reconstruction during OER.^[^ ^25^ ^]^ However, the exact oxidation states and coordination structures of the metal centers under operating conditions remained unclear.

This study aims to investigate the knowledge gap above by conducting operando XAS on a series of complex oxide catalysts with tunable degrees of surface reconstruction, and the hypothesis of achieving strong anticorrosion in direct seawater electrolysis (DSE) through surface reconstruction‐free behavior of designed electrocatalysts. The XAS studies confirm the reconstruction‐free behaviors of dual‐doped (i.e., silver‐ and cerium‐doped) iron manganese oxyhydroxide (AgCe‐FeMnOH). The electrocatalytic activity results show that AgCe‐FeMnOH can still exhibit exceptional OER activity without the need for surface reconstruction. By assembling AgCe‐FeMnOH into an anion exchange membrane (AEM) electrolyzer with an active electrocatalyst area to be 25 cm^2^, a highly durable alkaline DSE with an electrolyzing current of 5000 mA for at least 250 h at around 2.1 V (<4% potential decay) can be achieved. By comparison, the blank sample rapidly deactivates within 1 h. Our results suggest that surface reconstruction‐free characteristic can be a new crucial anti‐seawater corrosion strategy in addition to the widely adopted chloride‐repelling approach based on the hard–soft acid–base (HSAB) principle.

## 2 Results and Discussion

### 2.1 Sample Preparation

**Figure 1:**
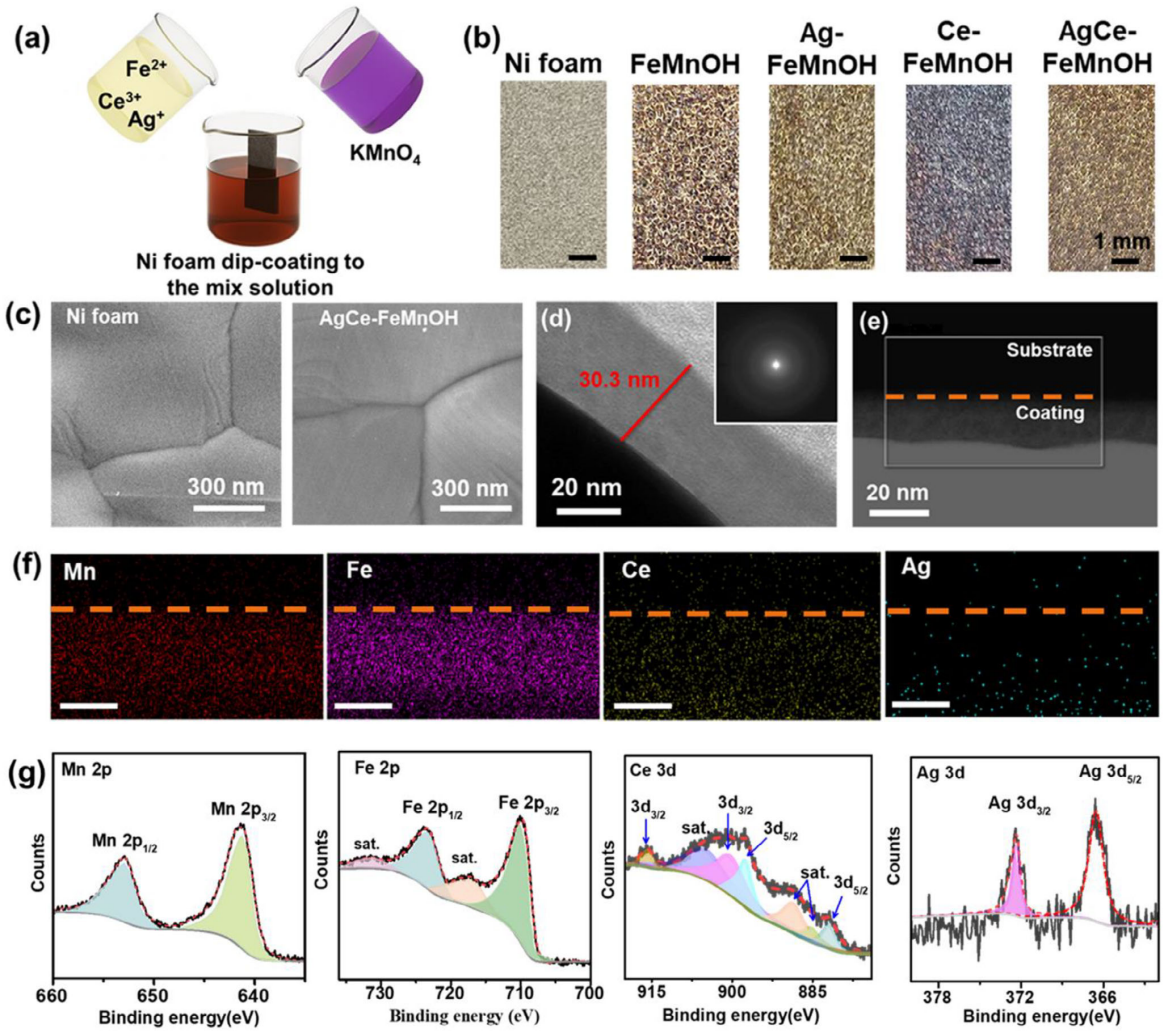
Synthesis and structural characterization of AgCe–FeMnOH electrocatalysts. a) Schematic illustration of the synthesis route for AgCe–FeMnOH complex oxide electrocatalysts deposited on Ni foam via the acidic redox‐assisted deposition (ARD) method. b) Photographs of Ni foam substrates coated with FeMnOH, Ag–FeMnOH, Ce–FeMnOH, and AgCe–FeMnOH. c) SEM image of the AgCe–FeMnOH film, showing a smooth surface morphology similar to that of the underlying Ni foam. d) High‐resolution TEM image of AgCe–FeMnOH, indicating a uniform film thickness of ≈30 nm; inset: selected area electron diffraction (SAED) pattern confirming the amorphous nature of the ARD products. e) High‐angle annular dark‐field scanning transmission electron microscopy (HAADF‐STEM) image and f) the corresponding elemental mapping of Fe, Ag, Mn, and Ce in AgCe–FeMnOH sample, revealing homogeneous elemental distribution; scale bars = 20 nm. g) XPS spectra of AgCe–FeMnOH thin film deposited on a silicon substrate, showing high‐resolution scans of Mn 2p, Fe 2p, Ce 3d, and Ag 3d. Experimental data are shown in dark gray, and fitted curves are overlaid in red dashed lines.

The amorphous AgCe‐FeMnOH oxyhydroxide electrocatalysts were prepared on Ni foam via a single‐step acidic redox‐assisted deposition (ARD) process^[^ ^26^, ^27^, ^28^ ^]^ by redox reaction of iron (II) sulfate and potassium permanganate (VII) followed by doping of cerium (III) nitrate and silver (I) nitrate at room temperature.^[^ ^25^ ^]^ The parent catalyst without dopants is named as iron‐manganese oxyhydroxide (designated as FeMnOH), while with one dopant samples are Ag‐doped FeMOH (Ag‐FeMnOH) and Ce‐doped FeMOH (Ce‐FeMnOH). Further, combining these two metals dopant, denoted as AgCe‐FeMnOH are prepared with 10 mol% (i.e., 3 mol% of Ag^I^ and 7 mol% of Ce^III^). This optimum ratio was achieved by systematically varied the Ag:Ce precursor ratio while keeping the total dopant concentration constant at 10 mol% (Ag + Ce). The resulting linear sweep voltammetry (LSV) revealed that this composition provides the highest OER activity (Figure S1, Supporting Information). Cerium has been recognized to utilize the redox couple of Ce^3+^/Ce^4+^ to stabilize the oxidation states of electrocatalysts.^[^ ^29^ ^]^ Silver (Ag) on the other hand has been reported to stabilize lattice oxygen, thereby minimizing undesirable surface reconstruction during OER.^[^ ^30^ ^]^ Doping these two metal cations should benefit our research scope. These samples were deposited on Ni foam by a 15 min solution process (see the illustration in **Figure** **1**a). The appearance of the deposition is shown in Figure 1b, while the elemental composites are shown in Table S1 (Supporting Information).

### 2.2 Material Characterization

The grazing incidence X‐ray diffraction (GIXRD) patterns (Figure S2, Supporting Information) show no diffraction peaks coming from the thin film AgCe‐FeMnOH, agreeing with the amorphous features and the earlier reports.^[^ ^27^ ^]^ The SEM images of AgCe‐FeMnOH show a smooth surface (Figure 1c), nearly identical to the surface feature of the primitive Ni foam. The high‐resolution transmission microscopy (HRTEM) of FIB‐sampled AgCe‐FeMnOH shows a complete film coverage, similar to the previous study on the ARD multimetal oxide film deposition,^[^ ^31^ ^]^ on Ni foam with thickness of 30.3 nm (Figure 1d). The selected area electron diffraction (SAED) patterns (inset in Figure 1d) further confirm the amorphous features of AgCe‐FeMnOH. The high‐angle annular dark‐field scanning transmission electron microscopy (HAADF‐STEM) images (Figure 1e) and the corresponding EDX mapping (Figure 1f, taken from the area in Figure 1e) show a uniform composition over the films, with a uniform distribution of Ag, Ce, Fe and Mn in the samples. Due to the lower contents of Ag and Ce in the samples, the elemental mappings of Auger electron spectroscopy (AES) were conducted and shown in Figure S3 (Supporting Information), where Ce and Ag dopants are evenly distributed over the entire AgCe‐FeMnOH samples.

The X‐ray photoelectron spectroscopy (XPS) results of AgCe‐FeMnOH (Figure 1g) show the high‐resolution spectra of Mn 2p, Fe 2p, Ce 3d, and Ag 3d. The high‐resolution Fe 2p spectra display the peaks at 712.0, 718.0, 724.6, and 730.5 eV assigned to Fe 2p_3/2_, the satellite peaks of 2p_3/2_, Fe 2p_1/2_, and the satellite peaks of 2p_1/2_, respectively—characteristic of Fe^3+^ species.^[^ ^32^, ^33^ ^]^ The Mn 2p spectra indicate the presence of Mn^3+^ and/or Mn^4+^ oxidation states, though a definitive distinction between them cannot be made based solely on XPS, consistent with previous reports.^[^ ^26^, ^34^, ^35^ ^]^ The Ce 3d spectra reveal multiple peaks at 881.9, 885.2, 889.9, 897.6, 901.0, 905.6, and 915.9 eV, including two prominent satellite peaks, which are collectively indicative of a dominant Ce^4+^ oxidation state. For Ag, the observed binding energies at 366.7 and 372.9 eV correspond to Ag 3d_5/2_ and Ag 3d_3/2_, consistent with metallic Ag^0^.^[^ ^36^ ^]^ The survey spectrum can be found in Figure S4a (Supporting Information). The O 1s spectrum (Figure S4b, Supporting Information) show both metal─O and metal─OH bonding, agreeing with that of metal oxyhydroxides.^[^ ^26^, ^37^ ^]^

The high‐resolution XPS analysis of Mn 2p and Fe 2p regions was also performed for undoped (FeMnOH) and single‐doped (Ag‐FeMnOH and Ce‐FeMnOH) samples to understand the effect of silver and cerium doping on the surface composition and electronic structure of FeMnOH (Figure S5, Supporting Information). Compared to the undoped FeMnOH, both Ag‐ and Ce‐containing samples exhibit a small positive shift (≈0.1–0.3 eV) in the Mn 2p_3/2_ and Fe 2p_3/2_ peaks. These shifts indicate a slightly higher average oxidation state of Mn and Fe after incorporation of Ag and Ce, suggesting that the dopants induce charge redistribution within the oxyhydroxide lattice.

### 2.3 Operando X‐Ray Absorption Spectroscopy

**Figure 2:**
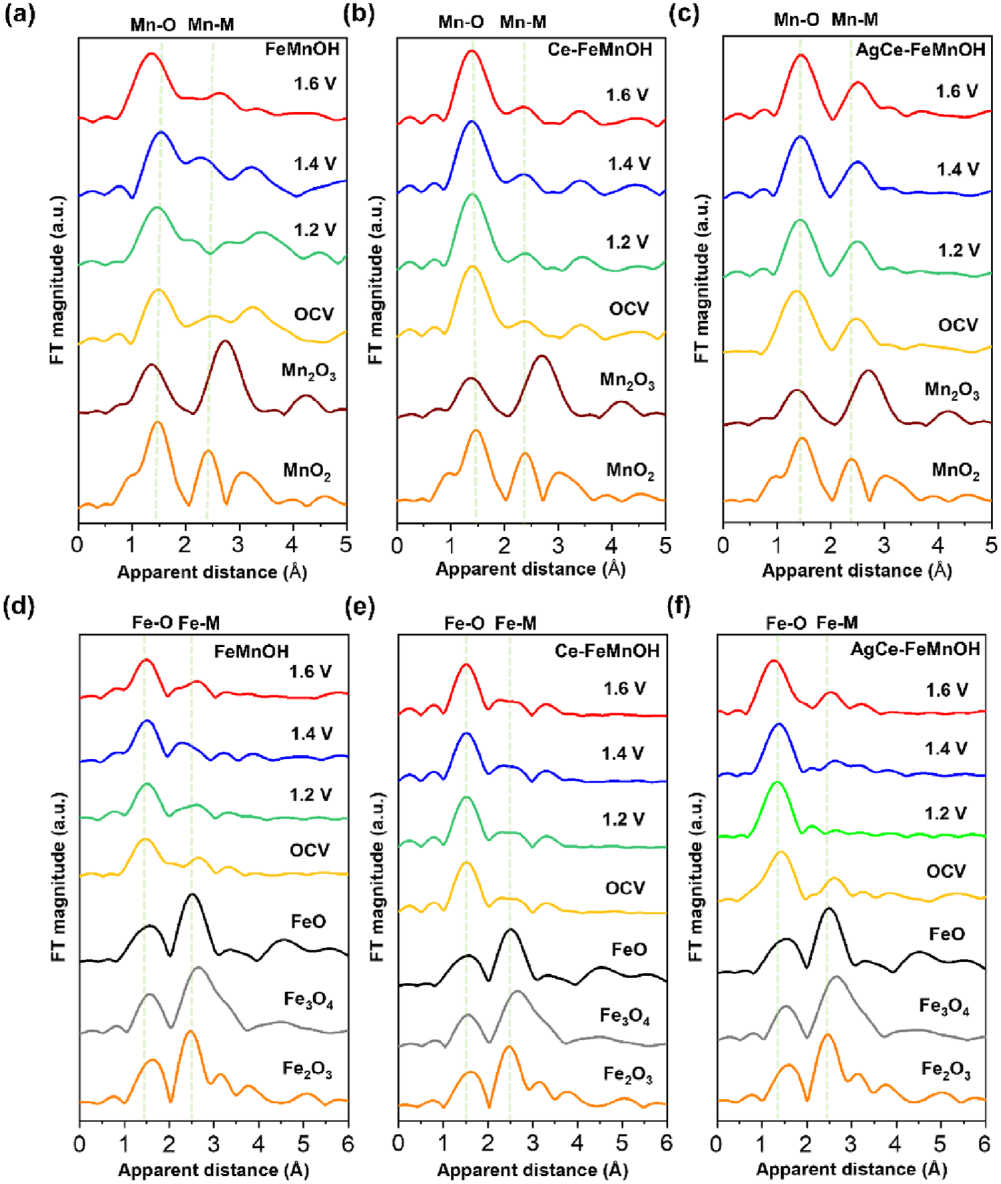
Operando XAS measurements illustrating the structural evolution of Fe‐ and Mn‐based catalysts under applied potentials. k^3^‐weighted Fourier‐transformed EXAFS spectra at the a–c) Mn K‐edge and d–f) Fe K‐edge are shown for a,d) FeMnOH, b,e) Ce‐FeMnOH, and c,f) AgCe‐FeMnOH, respectively. All measurements were conducted in 0.1 m KOH + 1 M NaCl using a Pt counter electrode and an Ag/AgCl reference electrode.

Given that Fe and Mn are the primary metallic components of the catalyst, we conducted an in‐depth investigation into their chemical valence states and coordination structures. While previous XPS analyses provided initial insights, they are insufficient to completely verify the true active structures under practical catalytic conditions—namely, during the oxygen evolution reaction (OER) in a saline alkaline electrolyte (0.1 m Fe‐free KOH with 1 m NaCl). Therefore, operando XAS at the Mn and Fe K‐edges was employed to capture the real‐time structural evolution of the catalysts under operating conditions. **Figure** **2** presents the operando k^3^‐weighted Fourier transformed extended X‐ray absorption fine structure (EXAFS) spectra of the Mn K‐edge (Figure 2a–c) and Fe K‐edge (Figure 2d–f). The Mn─O coordination structure of FeMnOH more closely resembles that of high‐valent MnO_2_, as evidenced by its shorter Mn─O bond distances and more symmetric first‐shell coordination, suggesting a higher average oxidation state of Mn in the pristine material (Figure 2a). In contrast, Ce‐FeMnOH exhibits Mn─O bond characteristics more similar to those of Mn_2_O_3_, featuring slightly longer Mn─O distances and more distorted local geometry (Figure 2b). These distinctions imply that Ce doping modulates the electronic environment around Mn, which may in turn influence the catalytic behavior and structural stability of the material under OER conditions. Compared to reference manganese oxides such as Mn_2_O_3_ and MnO_2_, both FeMnOH and Ce‐FeMnOH exhibit lower Mn–M coordination strength, indicating weaker metal–metal interactions in their bulk structures. This weakened coordination is particularly pronounced in FeMnOH, where the Mn coordination environment becomes distinctly unstable under higher applied potentials during electrochemical operation. These observations suggest that the incorporation of Ce dopants may exert a synergistic effect in stabilizing the Mn coordination structure. Furthermore, the dual doping in AgCe‐FeMnOH appears to preserve the original Mn coordination in the all applied potential conditions (Figure 2c), implying enhanced structural robustness of the Mn centers compared to the undoped or single‐doped systems.

After elucidating the dynamic structural evolution of Mn coordination environments during the electrochemical process, we further extended our operando investigations to the Fe centers to gain a more comprehensive understanding of their behavior under OER conditions. The undoped FeMnOH samples show a clear modulation in both Mn and Fe EXAFS signals upon increasing the potentials, indicating dynamic changes in the local coordination environments. This suggests the occurrence of electrochemically driven reconstruction processes involving changes in bond distances and coordination numbers (Figure 2d). Except for FeMnOH, all doped systems—including Ce‐FeMnOH and AgCe‐FeMnOH—exhibit minimal structural changes in the Fe local coordination environment under applied potentials up to 1.6 V, demonstrating the stabilizing effects of Ce and Ag dopants in harsh electrolytic environments (Figure 2e,f). These data show a good agreement with the previous in situ Raman data.^[^ ^25^ ^]^ Notably, in the AgCe‐FeMnOH sample (Figure 2f), a clear shortening of the Fe─O bond length is observed at elevated potentials, which is indicative of the formation of high‐valent Fe═O species and suggests that Fe may serve as an additional active center during the OER.^[^ ^38^ ^]^ Despite this transformation in the first‐shell Fe─O coordination, the Fe–M second‐shell distances remain essentially unchanged, with no evident peak shift or loss in intensity. This stability confirms the preservation of the metal–metal framework, supporting a surface reconstruction‐free scenario in which the active site geometry remains intact even under oxidative stress. These results underscore the synergistic role of Ce and Ag in not only stabilizing the Fe coordination structure but also enabling the formation of catalytically active Fe intermediates without compromising bulk structural integrity. This stabilization effect could have significant implications for improving catalyst durability and maintaining activity over prolonged operation, especially in seawater electrolysis environments where chloride‐induced degradation is a concern.

To fully understand the structural insights obtained from EXAFS results, operando XANES measurements of FeMnOH, Ce‐FeMnOH, and AgCe‐FeMnOH were conducted under identical electrochemical conditions (Figures S6 and S7, Supporting Information). For the Mn K‐edge (Figures S6a–c and S7a–c, Supporting Information), all the Mn sites exhibit negligible edge shifts as the applied potential increases, indicating that the oxidation state of Mn remains highly stable throughout the electrochemical operation. Compared with standard references, the Mn K‐edge position is consistent with Mn^4+^ species, which helps clarify the ambiguity in oxidation state assignment from the earlier XPS analysis. The discrepancy between XPS and XANES results can be attributed to the fundamental differences between the techniques: XPS probes the surface (≈5–10 nm) under ex situ conditions, whereas XANES captures the average oxidation state of the bulk under operando conditions. In addition, the pre‐edge feature remains unchanged across all applied potentials, suggesting that the local Mn coordination symmetry and ligand field remain largely preserved during electrochemical oxidation.

**Figure 3:**
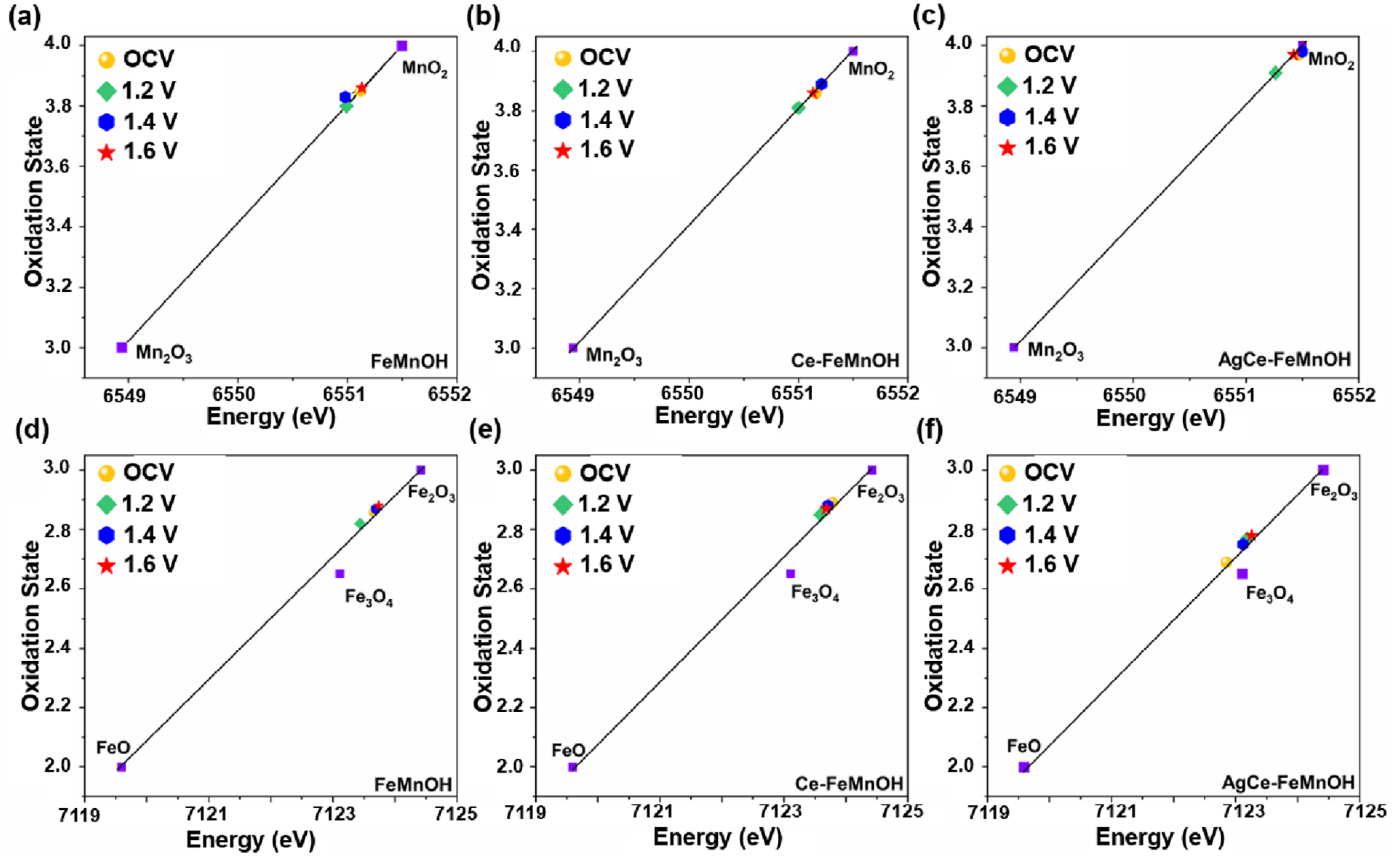
The corresponding oxidation number based on the linear fitting of the absorption edge position of XANES spectra. Panels a–c) show Mn K‐edge XANES spectra for FeMnOH, Ce‐FeMnOH, and AgCe‐FeMnOH, respectively, while d–f) display the corresponding Fe K‐edge spectra. All the samples were measured under alkaline conditions of 0.1 m KOH + 1 m NaCl with Pt as a counter electrode and Ag/AgCl reference electrode.

By referencing standard compounds and conducting linear fitting of the absorption edge positions, the corresponding Mn oxidation states can be semi‐quantitatively estimated (**Figure** **3**a–c). The analysis reveals that the Mn in FeMnOH is already close to a +4 oxidation state even under open‐circuit conditions and does not evolve significantly under applied potentials. Upon introducing Ce (Ce‐FeMnOH), the Mn oxidation state becomes slightly more positive, trending further toward Mn^4+^. This effect is even more pronounced in the AgCe‐FeMnOH system, where the edge position nearly overlaps with that of MnO_2_, indicating that Mn is stabilized in a high‐valent Mn^4+^ state. Additionally, the unchanging pre‐edge feature across all potentials supports the idea that Mn maintains an octahedral coordination geometry (Figure S6, Supporting Information), further reinforcing the role of Ce and Ag in preserving the active Mn framework under electrochemical stress.

The estimated Fe oxidation states derived from linear fitting of the Fe K‐edge absorption edge positions (Figures S6d–f and S7d–f, Supporting Information) reveal that, across all three samples—FeMnOH, Ce‐FeMnOH, and AgCe‐FeMnOH—the Fe centers lie within the mixed‐valence range of +2 to +3 under electrochemical conditions (Figure 3d–f). This observation aligns with the expected redox flexibility of Fe‐based catalysts during the OER. Notably, AgCe‐FeMnOH (Figure 3f) exhibits a more pronounced shift in oxidation state relative to OCV with increasing potential, approaching values close to Fe^3+^ at 1.6 V (vs RHE). It also provides direct evidence that complements the Fe─O bond contraction observed in the EXAFS data—suggesting the formation of high‐valent Fe═O moieties as catalytically relevant intermediates. These findings highlight the unique redox behavior of AgCe‐FeMnOH, where Ce‐doping stabilizes the overall metal coordination framework and co‐doping it with Ag further facilitates the dynamic formation of highly oxidized Fe centers, that might contribute to the superior OER performance and durability in alkaline seawater.

Taken together, the results indicate that the role of Mn is a more passive structural component during NaCl‐containing OER.^[^ ^39^ ^]^ In contrast, Fe centers exhibit dynamic redox behavior, with oxidation states ranging from +2 to +3 under OER‐relevant conditions. Among the samples, AgCe‐FeMnOH displays a more substantial potential‐dependent edge shift. The dopants of Ce and Ag are not altering Mn redox behavior but instead influencing the Fe environment or the overall lattice stability.

It is important to distinguish the local redox dynamics and structural reconstruction. Our XANES and EXAFS analyses show that Mn maintains an invariant oxidation state and coordination environment, while for Fe, a slight contraction in Fe─O distance and modest oxidation shift are observed at higher anodic bias. We attribute these slight variations to the redox activity of Fe sites, which function as the catalytic centers during OER. Such local electronic and bond‐length changes are expected for catalytic activity and differ fundamentally from surface reconstruction, which involves irreversible lattice rearrangement, phase conversion, or dissolution/redeposition processes. The lack of Fe─M coordination loss and the spectroscopically stable Mn environment therefore supports the classification of AgCe–FeMnOH as “reconstruction‐free,” while acknowledging that Fe undergoes intrinsic local redox dynamics associated with its catalytic role.

In this study, we focused operando XAS on Fe and Mn, as they are the main components of the amorphous framework and show the key redox/coordination changes linked to reconstruction resistance. The dopants, Ag and Ce in the catalyst (Ag:Ce:Fe:Mn = 0.01:0.22:2.96:1), are minor stabilizing components, and their low content makes operando L‐edge XAS technically impractical in operando alkaline cells due to strong attenuation and weak fluorescence signals. The mechanistic conclusions are governed by the Fe/Mn framework, supported by operando XAS, faradaic selectivity, stability, and absence of metal leaching. This conclusion is further confirmed by the ex‐situ XPS of AgCe‐FeMnOH before and after OER stability (Figure S8, Supporting Information) presented in the following section. Future work could employ more sensitive approaches, such as ambient‐pressure XPS to directly probe Ag^0^ and Ce^3+^/Ce^4+^ dynamics under bias.

### 2.4 OER Performance and Anticorrosion Behavior in Alkaline Seawater

**Figure 4:**
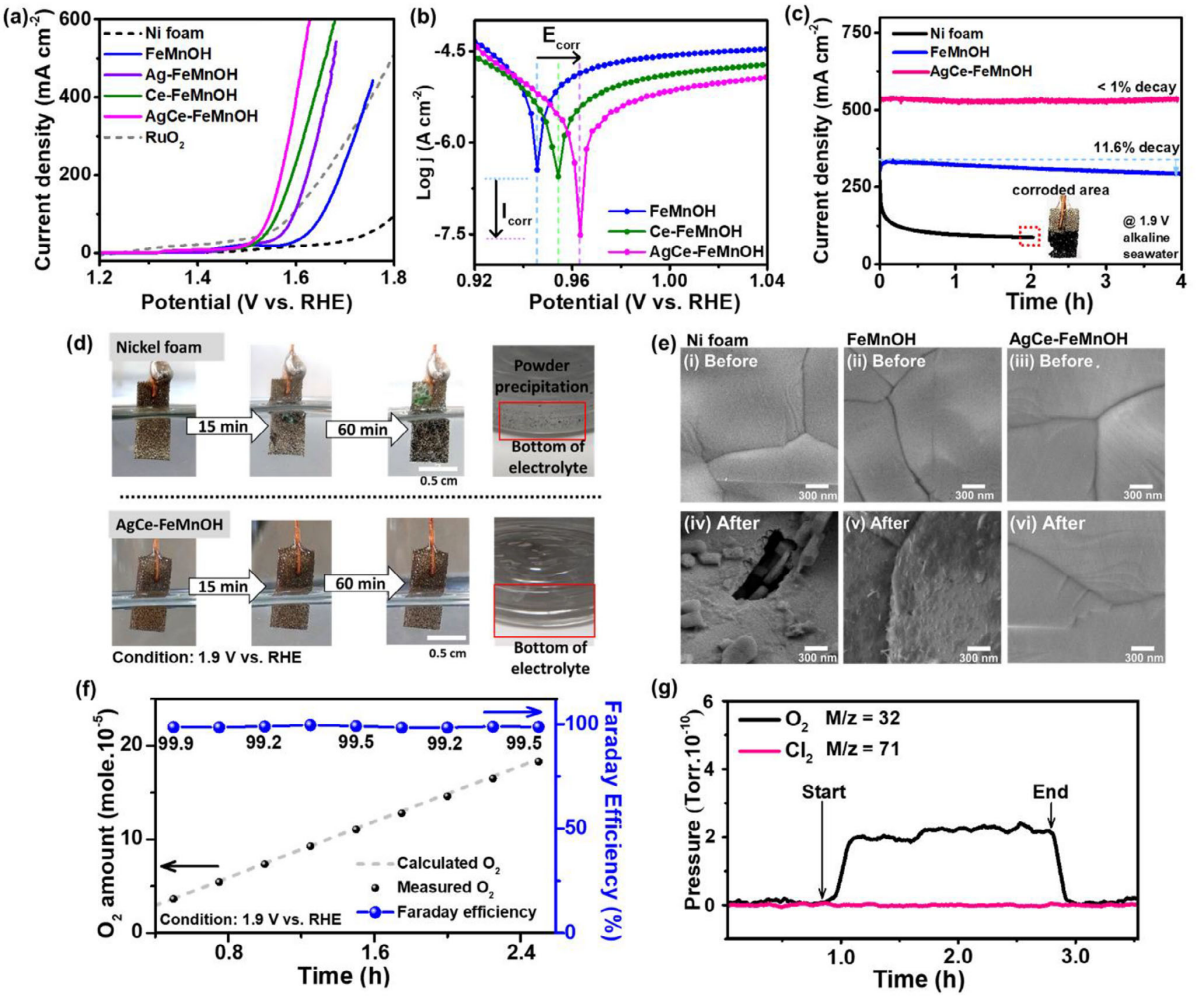
Electrocatalytic performance and corrosion resistance of FeMn‐based catalysts in alkaline seawater (1 m KOH seawater) using a three‐electrode setup. a) Linear sweep voltammetry (LSV) curves comparing OER activity of FeMnOH, Ag‐FeMnOH, Ce‐FeMnOH, and AgCe‐FeMnOH, all supported on Ni foam. b) Tafel plots show corrosion behavior, where AgCe‐FeMnOH exhibits the lowest corrosion current density (*i*_corr_) and highest corrosion potential (*E*_corr_), indicating superior anti‐corrosion properties against seawater. c) Chronoamperometry at 1.9 V (vs RHE) showing long‐term stability, with AgCe‐FeMnOH maintaining >99% current retention, while FeMnOH shows appreciable current decay and bare Ni foam rapidly fails due to corrosion. d) Digital images showing visual corrosion in the first 1 h at 1.9 V. Bare Ni foam displays surface degradation and brown precipitate formation in the alkaline seawater electrolyte, while AgCe‐FeMnOH remains visually intact without powder formation. e) SEM images of Ni foam, FeMnOH, and AgCe–FeMnOH before (i–iii) and after (iv–vi) electrolysis. Post‐OER, bare Ni foam exhibits severe surface damage, FeMnOH displays moderate roughening, and AgCe–FeMnOH preserves a smooth, unchanged morphology, confirming its outstanding corrosion resistance. All the electrochemical tested here is using Pt plate as the counter electrode and Hg/HgO as the reference electrode. f) Faradaic efficiency for oxygen evolution of AgCe‐FeMnOH closely matches theoretical values of oxygen evolution. g) The in situ, real‐time detection of the anode gas products using mass spectrometry during the OER operation in 1 m KOH seawater electrolyte. The partial pressures of O_2_ (black line, m/z = 32) and Cl_2_ (pink line, m/z = 71) were monitored. Upon application of anodic potentials (marked “Start”), an increase of O_2_ signal was observed, in contrast, no detectable Cl_2_ gas (no changes to the baseline) throughout the entire measurement period, confirming the selective OER activity and suppression of the chlorine evolution under the seawater electrolysis conditions.

To evaluate the electrocatalytic activity and corrosion resistance of the FeMn‐based catalysts under seawater‐relevant conditions, OER performance tests were carried out in a three‐electrode system using alkaline seawater electrolyte (1 m KOH seawater). The results are summarized in **Figure** **4**.

As shown in the polarization curves (Figure 4a), the AgCe‐FeMnOH catalysts show the highest OER activity among the series, achieving lower overpotential at a given current density compared to the undoped FeMnOH and single‐doped Ce‐FeMnOH or Ag‐FeMnOH. For example, at the current density response of 10 and 100 mA cm^−2^, AgCe‐FeMnOH requires only a small overpotential of 210 and 320 mV, respectively. At overpotential of 400 mV, the AgCe‐FeMnOH delivers a current density up to 600 mA cm^−2^, which is about fourfold higher than the benchmark RuO_2_. The overall trend in activity of AgCe‐FeMnOH > Ce‐FeMnOH > FeMnOH (similar to the activity and activation energy trend under NaCl‐free alkaline conditions)^[^ ^25^ ^]^ providing further support for the finding observed in XANES, that co‐doping Ag with Ce leads to the dynamic formation of highly oxidized Fe centers, while for the structural stability observed in EXAFS: AgCe‐FeMnOH ≈ Ce‐FeMnOH > FeMnOH. This consistency highlights that exceptional OER activity does not require surface reconstructions, both under alkaline freshwater and seawater OER.

The Tafel corrosion plots (Figure 4b) were used to evaluate the corrosion resistance of the catalysts in alkaline seawater. Among all the samples, AgCe‐FeMnOH exhibits the most favorable corrosion parameters, with the highest corrosion potential (*E*_corr_ = 0.964 V) and the lowest corrosion current density (*I*_corr_ = 1.92×10^−6 ^mA cm^−2^), indicating enhanced resistance to electrochemical degradation. In contrast, FeMnOH shows *E*_corr_ = 0.947 V and *I*_corr_ = 6.96×10^−6 ^mA cm^−2^, while Ce‐FeMnOH displays intermediate values (*E*_corr_ = 0.955 V; *I*_corr_ = 4.55×10^−6 ^mA cm^−2^). These results confirm that AgCe‐FeMnOH possesses the highest corrosion resistance among the tested catalysts.

To further evaluate the impact of surface reconstruction‐free behavior on the anti‐corrosion performance, we conducted stability tests of the selected catalysts at a fixed potential of 1.9 V (Figure 4c). AgCe‐FeMnOH, as the representative of surface reconstruction‐free sample, exhibits highly stable current output (i.e., less than 1% current density drop) over the extended operation, at least for 4 h, indicating excellent durability in alkaline seawater. In contrast, the FeMnOH electrode, which still exhibits certain degrees of surface reconstruction behaviors, shows a significant current drop followed by a progressive decay (>11%). Bare Ni foam exhibits even faster deterioration characterized by rapid loss of activity within the first hour, agreeing with the test results above.

The trends of the durability tests align well with those of the operando XAS results, the less degrees of surface reconstruction leading to enhanced anticorrosion properties. The absence of performance decay implies that the catalyst remains chemically stable under continuous OER condition and resists Cl^−^ attack even at elevated anodic potentials.

Further visual evidence of corrosion resistance is provided in Figure 4d, complementing the stability assessment in Figure 4c. The physical appearance of the electrodes after 1 h of operation at 1.9 V (vs RHE) in alkaline seawater reveals striking differences. The AgCe‐FeMnOH‐coated Ni foam maintained a clean, metallic surface with no visible signs of corrosion or discoloration. In contrast, the bare Ni foam exhibited significant surface darkening and degradation within the first 15 min, indicative of rapid chloride‐induced corrosion. Notably, a black precipitate was observed in the electrolyte surrounding the bare Ni foam, likely resulting from the dissolution and redeposition of nickel species. These precipitates may primarily consist of Ni(OH)_2_, NiO, or NiCl_2_·*x*H_2_O, formed via anodic oxidation in the presence of Cl^−^ and OH^−^ ions.^[^ ^40^ ^]^ Such degradation byproducts were absent in the case of AgCe‐FeMnOH, further highlighting its superior stability. Ex situ XPS analysis (Figure S8, Supporting Information) further confirms the stability of the catalyst (post‐OER analysis). While the dopants, Ag remains metallic (Ag^0^) with no chemical environment change, and Ce maintains its mixed Ce^3+^/Ce^4+^ states with only minor shifts. The observed shifts in Ce 3d spectra reflect the redox flexibility of Ce. The dominant metal compositions, Mn oxidation remains unchanged, and Fe shows only slight variations consistent with operando XAS. The preservation of metal dopants, especially Ce, reflects the inherent redox flexibility of Ce, where Ce can reversibly between +3 and +4 oxidation states while maintaining structural stability. These results indicate that the stability of AgCe‐FeMnOH under alkaline seawater OER conditions.

To establish the relationship between seawater anticorrosion performance and surface reconstruction behavior, we further examined morphological changes using SEM. It is well‐documented that electrocatalysts undergoing surface reconstruction typically exhibit noticeable morphological alterations under SEM, as reported in the previous studies.^[^ ^41^, ^42^ ^]^ In the SEM results (Figure 4e), FeMnOH shows significant surface morphology changes after electrolysis, consistent with the minor reconstruction behavior previously reported in the literature and confirmed by our XAS analysis. In contrast, AgCe‐FeMnOH displays no discernible changes in SEM before and after electrolysis, providing direct evidence of its reconstruction‐free nature. Additionally, the bare Ni foam surface reveals severe surface damage and structural disintegration after the stability tests in seawater, confirming the critical role of the complex oxide coatings, particularly AgCe‐FeMnOH, providing an effective barrier against seawater corrosion. To further assess the structural stability of AgCe‐FeMnOH, HRTEM was conducted on AgCe‐FeMnOH powder prepared under identical conditions. As shown in Figure S9 (Supporting Information), both pre‐ and post‐OER (the same OER stability as Figure 4c) samples exhibit an amorphous structure, with no visible lattice fringes and SAED patterns displaying only diffuse halos. These results confirm that the amorphous nature of AgCe‐FeMnOH is preserved during alkaline seawater OER, without evidence of lattice rearrangement or crystallization. Taken together, these observations strongly support the conclusion that the exceptional corrosion resistance of AgCe‐FeMnOH in alkaline seawater arises from its ability to maintain a stable, reconstruction‐free surface under harsh electrochemical conditions.

The Faradaic efficiency measurements (Figure 4f) associated with the tests above in the alkaline seawater electrolysis confirm that the evolved oxygen closely matched the theoretical value (Faraday efficiency ≈ 99.5%), further supporting that nearly all the provided charge/electricity contributed to oxygen production. Based on the results of real‐time gas detection during the electrolysis in alkaline seawater (Figure 4g), no Cl_2_ gas can be detected, while oxygen gas was clearly produced at the same time. This finding suggests that the electricity is selectively conducting toward OER rather than being consumed by the chlorine evolution or other corrosion‐derived side reactions.

### 2.5 AEM Electrolyzer Performance and the Role of Reconstruction‐Free Stability

**Figure 5:**
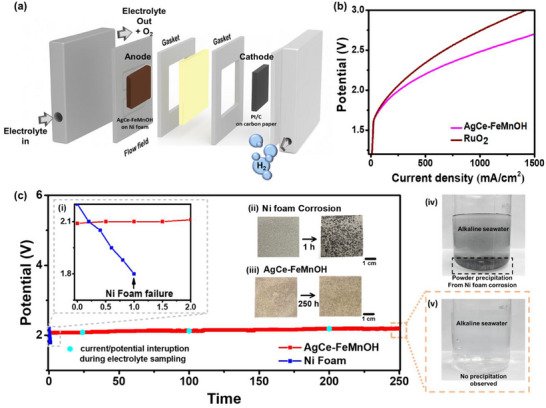
Performance and corrosion resistance of AgCe‐FeMnOH anode in a zero‐gap anion exchange membrane (AEM) electrolyzer using alkaline seawater. a) Schematic illustration of the AEM electrolyzer configuration. Electrodes (5 × 5 cm^2^) were assembled with Teflon gaskets (matching electrode thickness) as spacers and titanium flow fields. Electrolyte (2 m KOH + natural seawater) was circulated only on the anode side, while the cathode side remained dry. The anode consisted of AgCe‐FeMnOH coated on Ni foam, and the cathode used commercial Pt/C on carbon paper. b) Polarization curves comparing the performance of AgCe‐FeMnOH and commercial RuO_2_ as OER electrocatalysts in alkaline seawater. AgCe‐FeMnOH achieves higher current densities at equivalent electrolysis voltages. c) Constant‐current stability test at 5000 mA (equivalent to 200 mA cm^−2^) at least for 250 h at 50 °C. AgCe‐FeMnOH maintains a stable electrolysis voltage throughout, while blank Ni foam suffers rapid voltage increase and mechanical failure within 1 h due to chloride‐induced corrosion, as shown in panel (i). Post‐electrolysis images: (ii) severely corroded Ni foam after 1 h in seawater electrolysis, (iii) intact AgCe‐FeMnOH surface after 250 h in seawater electrolysis. Photographs of the electrolyte after the testing show (iv) visible black precipitate yielded from the blank Ni system, while (v) the AgCe‐FeMnOH electrolyzer system shows clean and transparent electrolyte without precipitation, indicating a corrosion‐free situation. The light blue dots in Figure 5c along the AgCe‐FeMnOH stability are the tracking of current/potential interruption by pausing/terminating the anodic bias during the operation, for electrolyte sampling. Despite the interruptions (at 24, 100, and 200 h), the AgCe‐FeMnOH electrode still exhibits exceptional stability without degradation (no metal leaching detected, Table S3, Supporting Information, ICP).

To evaluate the practical applicability of the AgCe‐FeMnOH catalyst against seawater corrosion and to realize DSE, zero‐gap system testing was performed using a 5 × 5 cm^2^ AEM electrolyzer under alkaline seawater conditions (2 m KOH seawater) as suggested in the literature.^[^ ^43^ ^]^ The AEM electrolyzer assembly (**Figure** **5**a) and operation introduction are included in the Supporting Information. As shown in Figure 5a, the AgCe‐FeMnOH anode, paired with a Pt/C on carbon paper cathode for hydrogen evolution, outperformed the benchmark RuO_2_ catalyst in terms of current response under identical conditions. This result confirms that the dual‐doped catalyst not only exhibits excellent intrinsic activity at the laboratory level (Figure 5b), but also scales up effectively in realistic electrolyzer applications.

The long‐term durability of the catalyst was further assessed by applying a constant current of 5000 mA (equivalent to a current density of 200 mA cm^−2^) in alkaline seawater electrolyte. As shown in Figure 5c, the AgCe‐FeMnOH electrode maintained a stable electrolysis voltage (at 2.1 V) throughout the entire operation, demonstrating excellent resistance to degradation under the high electrolysis current (see a comparison table of Table S2, Supporting Information, of the reported AEM electrolyzer operation). In contrast, the control study of an electrolyzer that used a bare Ni foam as the anode shows a rapid failure within 1 h of electrolysis, as shown in the inset photograph of Figure 5c‐i. When opening the electrolyzer, significant corrosion with blackened areas on the Ni foam anode can be clearly observed (Figure 5c‐ii). The appearance of AgCe‐FeMnOH after the 250 h of operation (Figure 5c‐iii) is nearly identical to that before the 250 h tests. No visibly noticeable changes or blackening can be seen.

This electrolyzer degradation can also be identified by the appearance of the seawater electrolyte. A significant formation of black precipitates can be observed for the anode cases of using bare Ni foam above, is clearly associated with the corrosion and precipitate formation seen in Figure 5c‐iv,v, and further emphasizes the challenge of chloride‐induced failure in alkaline DSE.

To further evaluate durability, we introduced current interruptions at 24, 100, and 200 h during the AgCe‐FeMnOH stability test, as indicated by the blue dots in Figure 5c. This approach simulates structural stress by alternating between the charged (bias‐on) state—often associated with surface reconstruction in conventional electrocatalysts—and the non‐charged (bias‐off) state.^[^ ^44^ ^]^ Each interruption cycle potentially induces elemental leaching and irreversible structural degradation. Despite this, the AgCe‐FeMnOH‐based AEM electrolyzer maintains stable operation with less than 4% voltage decay, underscoring the robustness of its reconstruction‐free nature. Supporting this, post‐test ICP analysis shows no detectable increase in Fe or Mn ion concentrations in the electrolyte (Table S3, Supporting Information), confirming the absence of catalyst dissolution.

Compared to previously reported AEM electrolyzer systems (Table S2, Supporting Information), our device employs a significantly larger electrocatalyst area (25 cm^2^) while maintaining excellent durability. Scaling up electrode area typically introduces challenges in achieving uniform catalyst coverage; any exposed regions on the Ni foam substrate are highly vulnerable to rapid corrosion under seawater electrolysis conditions. The stable performance observed here highlights the excellent homogeneity and scalability of AgCe–FeMnOH coatings prepared via the ARD method.

## 3 Discussion

In our earlier work, operando Raman spectroscopy suggested that AgCe‐FeMnOH maintains its structural integrity without undergoing significant surface reconstruction during the OER. In the present study, we further validate this observation using operando XAS, which reveals no detectable shifts in the oxidation states or local atomic structure across the OER‐relevant potential range. The combined spectroscopic evidence provides compelling support that AgCe‐FeMnOH remains reconstruction‐free under operating conditions.

These findings indicate an alternative OER pathway different from the prevailing one associated with the lattice oxygen mechanism, which posits that activation of lattice oxygen—often coupled with irreversible structural transformations—is necessary for enhanced OER activity. Our operando XAS results show preservation of both Mn and Fe local frameworks, with no evidence of lattice oxygen consumption or destabilization. We therefore consider that LOM mechanism can unlikely accounts for the catalytic behaviors in AgCe‐FeMnOH system.

On the other hand, the AEM (adsorbate evolution mechanism) pathway often begin with a crystalline oxide that reconstructs into amorphous M─OOH under operation, while AgCe‐FeMnOH is already in an amorphous M─OOH state prior to OER and shows no further transformation during electrolysis, suggesting that this AEM mechanism is also not can fully suitable pathway here. Taken together, we can only logically suggest that AgCe‐FeMnOH operates through a nonclassical OER pathway. Further studies such as oxygen isotope experiments may be help to clarify the actual mechanism pathway.

The mechanistic origin of this reconstruction‐resistant behavior remains elusive. We propose that it may depend on the number of dopants, wherein increased compositional complexity suppresses atomic rearrangements, or arise from a dopant‐specific effect, in which the chemical nature of Ag and Ce uniquely stabilizes the catalytic matrix. Disentangling these effects will require further systematic investigation. These studies also rely on the experimental success on precisely controlling the contents of each dopant. Future studies, both experimental and theoretical, are essential to advance a deeper understanding of the structural stability mechanisms in multinary oxide electrocatalysts.

The absence of surface reconstruction is responsible for the resistance to seawater‐induced corrosion. Comparative analysis between AgCe‐FeMnOH and FeMnOH, together with the SEM study of Ni foam corrosion in Figure 4e, provides experimental support for this correlation. Comparative analysis between AgCe‐FeMnOH and FeMnOH provides experimental support for this correlation. While FeMnOH undergoes partial surface reconstruction during OER, it also shows measurable degradation and surface deterioration under simulated seawater conditions (see Figure 4c, the durability tests). In contrast, AgCe‐FeMnOH displays reconstruction‐free behaviors and negligible signs of corrosion in seawater electrolysis. These observations suggest that surface reconstruction either directly correlates, or indirectly through multiple unknown intermediate states, with the resistance to seawater‐induced corrosion. Utilization of lattice oxygen during OER is expected to generate surface vacancies, allowing both hydroxide and chloride ions to competitively coordinate with metal cation active sites. Once Metal–Cl bonds are formed, key intermediates of the corrosion cycle can emerge, thereby significantly increasing the risk of degradation in seawater environments.^[^ ^45^ ^]^

In contrast, by suppressing such reconstruction processes, the participation of lattice oxygen for OER should be significantly restricted, and thus minimize chloride accessibility to the active centers, thereby achieving superior seawater durability. These findings offer a compelling design guideline for seawater‐OER electrocatalysts achieving long durability.

Although the suppression of surface reconstruction correlates with enhanced corrosion resistance, we acknowledge that the present study, also suggested by the reviewer, has not directly quantified the impacts of degrees of suppressed surface reconstruction on seawater anticorrosion. The energetic tendency of chloride ion coordination to all the metal centers of the catalyst (i.e., Ag(I), Ce(III/IV), Fe(III), and Mn(IV)) is also important to be considered together for a clearer understanding. Future studies employing theoretical calculations of Cl^−^ attachment energy together with more in‐situ spectroscopic quantification will be continued to clarify the actual pathway of this causal relationship.

## 4 Conclusion

In summary, this study demonstrates that achieving surface reconstruction‐free stability is a viable strategy for developing highly anticorrosive electrocatalysts for seawater splitting. Our findings establish a direct correlation between the suppression of surface reconstruction and enhanced resistance to chloride‐induced degradation. Through operando XAS conducted in this work, complemented by our previous operando Raman analysis, we provide compelling and consistent evidence that AgCe‐FeMnOH retains its structural integrity under OER‐relevant conditions, without undergoing surface reconstruction. This structural robustness is closely associated with superior catalytic activity and exceptional corrosion resistance in simulated seawater environments. The convergence of high activity and stability in a reconstruction‐free catalyst challenges the conventional view that lattice oxygen participation—and the accompanying surface transformation—is necessary for effective OER performance. Together, these findings suggest a new design principle: electrocatalysts that intrinsically resist surface reconstruction are more capable of offering long‐lasting stability in seawater electrolysis, helping to enable sustainable hydrogen production from ocean‐based resources. Further studies are needed to clarify dopant‐specific effects from compositional complexity and to validate this strategy under real seawater electrolysis or industrial‐scale conditions. This work provides new design principles and insightful behaviors for the future development of DSE‐compatible electrocatalysts.

## Conflict of Interest

The authors declare no conflict of interest.

## Supporting information



Supporting Information

## Data Availability

The data that support the findings of this study are available on request from the corresponding author. The data are not publicly available due to privacy or ethical restrictions.
