# Supplementary material for: Surface Reconstruction‐Free Stability Achieving Highly Anticorrosive Seawater Splitting
Source: Adv Sci (Weinh). 2025 Oct 27;13(1):e16499. doi: 10.1002/advs.202516499 (PMC12767128; doi:10.1002/advs.202516499)
Supplement: Supplementary file 1 — Supporting Information [file ADVS-13-e16499-s001.docx]

**Supporting Information**

Surface Reconstruction-free Stability Achieving Highly Anticorrosive Seawater Splitting

*Yanita Devi^a,b^†, Ruspika Sundaresan^c^†, Tilahun Awoke Zegeye^a^, Guan-Hong Guo^a^, Mariel G. Tecson^a,b^, Wei-Chun Lin^d^,* *Yu-Cheng Shao^f^, Ching-Wei Tung^c,e^* and Chun-Hu Chen^a,b^**

*^a^ Department of Chemistry, National Sun Yat-sen University, Kaohsiung 80424, Taiwan*

*^b^Green Hydrogen Research Center, National Sun Yat-sen University, Kaohsiung 80424, Taiwan*

*^c^ Center for Environmental Sustainability and Human Health, Ming Chi University of Technology, New Taipei City*

*24301, Taiwan*

*^d^Department of Photonics, National Sun Yat-Sen University, Kaohsiung 80424, Taiwan*

*^e^ Department of Materials Engineering, Ming Chi University of Technology, New Taipei City 24301, Taiwan*

*^f^National Synchrotron Radiation Research Center, Hsinchu, 300092 Taiwan*

*†These authors contributed equally to this work: Yanita Devi, and Ruspika Sundaresan*

Corresponding author, Ching-Wei Tung, email address: [chingweitung@mail.mcut.edu.tw](file:///C:\Users\guess\Desktop\Manuscript%20Prepared\陳軍互\chingweitung@mail.mcut.edu.tw); Tel: 02-29089899 ext. 6314; Fax: 886-2-2908-4091. Chun-Hu Chen, email address: [chunhu.chen@mail.nsysu.edu.tw](mailto:chunhu.chen@mail.nsysu.edu.tw); Tel.: 886-7-525-2000 ext. 3943; Fax: 886-7-525-3908.

**Experimental section**

**Preparation of Ni foam coated amorphous complex oxide electrocatalysts**

All chemicals were used as received without further purification. For materials synthesis, amorphous AgCe-FeMnOH electrocatalyst was synthesized via acidic redox-assisted deposition (ARD) method following our previous works.^1-3^ 2 mmol of KMnO_4_ (J.T.Baker) was dissolved into 50 mL of DI water (resistivity: 18.2 MΩ.cm) and stirred for 5 min to form solution A. Secondly, 5.4 mmol of FeSO_4_ ^.^7H_2_O (99%, ACROS), and 0.42 mmol of Ce (NO_3_)_3_^.^ 6H_2_O (99.5%, ACROS) were dissolved in 100 mL of DI water and stirred for 4 min. 0.18 mmol of AgNO_3_ (99%, Sigma-Aldrich) was added and continued stirring for 1 min, form solution B. The solution A and solution B were mixed and stirred for 1 minute. For the substrates, we mainly used Ni foam received from Innovation Materials Co., Ltd. Ni foam was cleaned with DI water, ethanol, acetone, and isopropyl alcohol (IPA) under sonication for 10 min followed by exposure to O_2_ plasma (40 W) for 3 min. The resulting Ni foam was washed with 100 mL of HCl and DI water mixture (1:9 v/v) and then rinsed with DI water. In the deposition process, Ni foam was immersed vertically into the mixed solution and sonicated at room temperature for 15 min. The sample was then removed from the solution, washed with DI water and acetone several times, and dried in air before electrochemical testing. Undoped metal oxide (FeMnOH) and Ag-doped (Ag-FeMnOH) and Ce-doped (Ce-FeMnOH) metal oxides were synthesized through the same procedure as (Ag, Ce) dual-doped (AgCe-FeMnOH) metal oxide except that dopants (Ag and Ce) were absent correspondingly.

**Materials characterization**

The morphology and microstructure of samples were determined by scanning electron microscopy (SEM, FEI Inspect F50) and transmission electron microscopy (TEM, JEOL, JEM-2010) coupled with energy dispersive X-ray (EDX) spectroscopy. The post-OER SEM images were performed after chronoamperometry test of alkaline seawater (1 M KOH seawater) splitting (Figure 4c). The film thickness and crystallinity of the samples were collected using high-resolution field-emission high-resolution transmission electron microscope (HRTEM) by cutting the sample with Focused ion beam (FIB, Hitachi NX2000). The phase composition of samples was characterized by X-ray diffraction (Bruker D2 Phaser equipped with CuKα (λ =1.5418Å) radiation source). The crystallographic orientations of samples were characterized by Grazing incidence X-Ray diffraction (GIXRD) with Bruker D8-Discover with a CuKα X-ray source with grazing angle. XPS was recorded with a PHI 5000 VersaProbe I system (ULVAC-PHI, Japan) with Al Kα X-ray (25 W, 100 μm). The takeoff angle of the photoelectron was fixed at 45°, and a dual-beam charge neutralizer was used for charge compensation. The narrow scan spectra were collected with a pass energy of 23.5 eV. The energy step of the spectra is 0.2 eV. No sputtering was performed prior to analysis.The Auger electron spectroscopy (AES) elemental mapping of Ag and Ce were collected using HORIBA GDS850A. The elemental analysis was carried out with an inductively coupled plasma mass spectrometer (ICP-MS) using a THERMO-ELEMENT XR spectrometer. Samples were dissolved in a solution composed of a volume ratio of DI water: HNO_3_: H_2_O_2_ = 3:2:1 respectively. A series of external standards was used to calibrate the instrument using the relevant concentration range. The ICP measurement of the electrolyte before and after stability was measured as it is.

**Electrochemical measurements**

All electrochemical tests for OER were carried out in three electrode electrochemical setup connected to electrochemical analyzer (CHI 704E, CH Instruments, Inc.) at scan rate of 50 mV/s in 1 M KOH seawater. The seawater was collected from Xiziwan Beach, near National Sun Yat-Sen University, Kaohsiung City, Taiwan and let it stands for several days, mixed with KOH powder until certain concentration then used as the electrolyte. For the three-electrode system, the prepared deposited catalyst (0.5 x 0.5 cm^2^) was utilized as the working electrode whereas Hg/HgO and Pt plates were used as the reference electrode and the counter electrode, respectively. The potentials measured against Hg/HgO were converted to the reversible hydrogen electrode (RHE) according to the reference electrode calibration. For the performance comparison, the RuO_2_ powder was dispersed in a 2.15 g mixture of DI water and 2-propanol with a mass ratio of 1:3.43, then mixed with 5 wt% AEM ionomer solution and followed by sonication for 30 min. The resulting catalyst ink then was deposited using spray method on Ni foam substrate. All electrochemical tests were reported with iR-correction unless otherwise specified. The correction of electrode potential was carried out using cell ohmic drop (R) from electrochemical impedance spectroscopy (EIS) at high frequency as in the following equation.

$E_{iR-corrected}= E_{RHE}-iR$ (S1)

where *E_RHE_* and *E_iR-corrected_* are the measured and corrected potentials in V respectively, while *i* is measured current in A, and *R* is the resistance in Ω.

The overpotential (η) of OER was calculated by using the equation.^3^

${\eta=E}_{RHE}-$ 1.23 (S2)

Tafel corrosion polarization measurements were carried out using a conventional three-electrode configuration, where a platinum plate and a Hg/HgO electrode served as the counter and reference electrodes, respectively. Prior to the test, the sample (1 × 1 cm^2^) was immersed in 1 M KOH seawater to ensure a steady open circuit potential (OCP).

**In-situ XAS measurement**

The in-situ X-ray absorption spectra of catalysts at the Mn-edge (absorption edge at *E^0^* = 6,539 eV) and the Fe K-edge (absorption edge at *E^0^* = 7,112 eV) were collected at the 12B2 Taiwan beamline (Spring-8, Japan) of National Synchrotron Radiation Research Center (NSRRC) operated at an 8.0 GeV storage ring with a constant current of ~100 mA, using a Si (111) double-crystal monochromator for the incident-beam energy. The scan range was 6,912 to 8,012 for Fe K-edge, in which the energy was calibrated accordingly to the first inflection point of the absorption edge of metallic Mn and Fe foil. Athena software was used to extract the XAS data composed of X-ray absorption near edge spectra/extended X-ray absorption fine structure, including pre-edge and post-edge background subtraction, normalization with respect to edge height, and Fourier transformation.

**Membrane preparation and AEM electrolyzer assembly**

The membrane preparation and assembly were carried out based on the reported literature.^5^ Briefly, the commercial membrane (FAA-3-50, Fumasep, thickness = 50 µm) was soaked in 1 M KOH for 1 day to realize satisfactory OH^-^ ion conductivity before assembling the device.

The OER anode of AgCe-FeMnOH prepared using the same method mention above, with the area of Ni foam substrate to be 5 x 5 cm^2^. For the comparison, the RuO_2_ catalyst ink was sprayed also at 5 x 5 cm^2^. For HER electrode, Pt/C in carbon paper (Fuel Cell store) was used. All catalysts were directly assembled (see Figure 5a) without any pre-treatment. The electrolyte of 2 M KOH seawater was flow through 1/8-inch tube with peristaltic pump with flow rate of 50 mL/min only at anode while the cathode side is in dry condition (no electrolyte flow). The electrolyte was kept stable at 50^o^C using temperature controller with thermocouple connected to the AEM electrolyzer.

**Gas production and Faraday efficiency measurements**

The mass measurement of gaseous products was performed continuously during water electrolysis by using mass spectroscopy (Hiden HPR-20 R&D) in a closed system of customized beaker. The test was done in range of 30-60 min, under 1.9 V applied potential in 1 M KOH seawater. The generated gasses were measured in-situ by direct flow with argon gas as the carrier gas. The resulting gas pressure (torr) at a certain time was calculated using oxygen (m/z = 32) calibration (prepared by measuring the known amount of oxygen gas flow versus the pressure detected in mass spectroscopy) to generate the real flow rate of the oxygen gas produced. The Faraday efficiency was calculated using the measured gas production divided by the theoretical gas produced (Figure 4f). While chlorine gas (m/z = 71) was not detected in the same test, shown in Figure 4g.


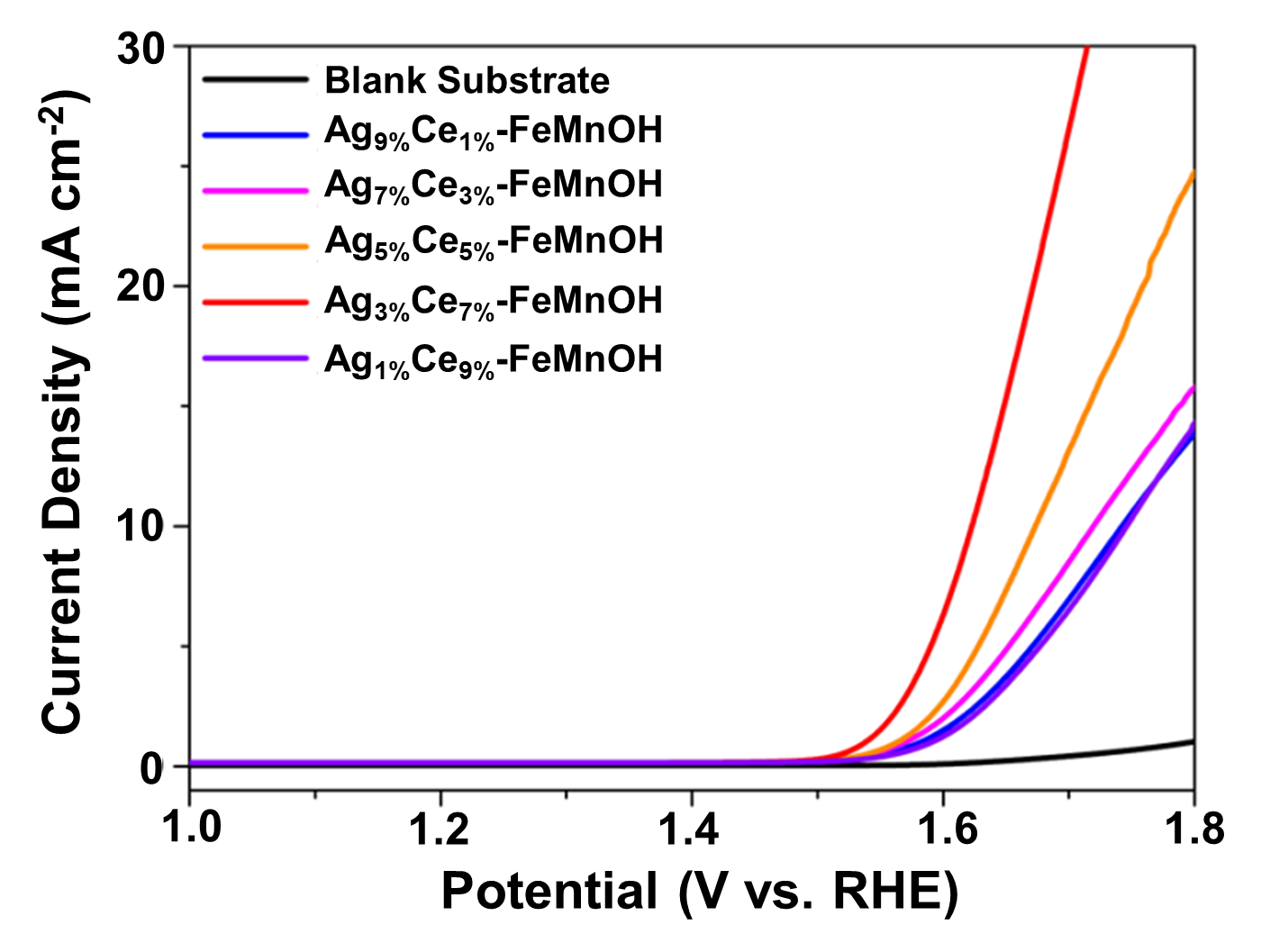


**Figure S1.** Linear sweep voltammetry (LSV) curves of FeMnOH thin-film electrocatalysts co-doped with different Ag and Ce ratios, maintaining a constant total dopant concentration of 10 mol% (Ag + Ce). The Ag 3%-Ce 7% sample (red curve) exhibited the highest OER activity, selected as the optimal doping ratio used in this study.


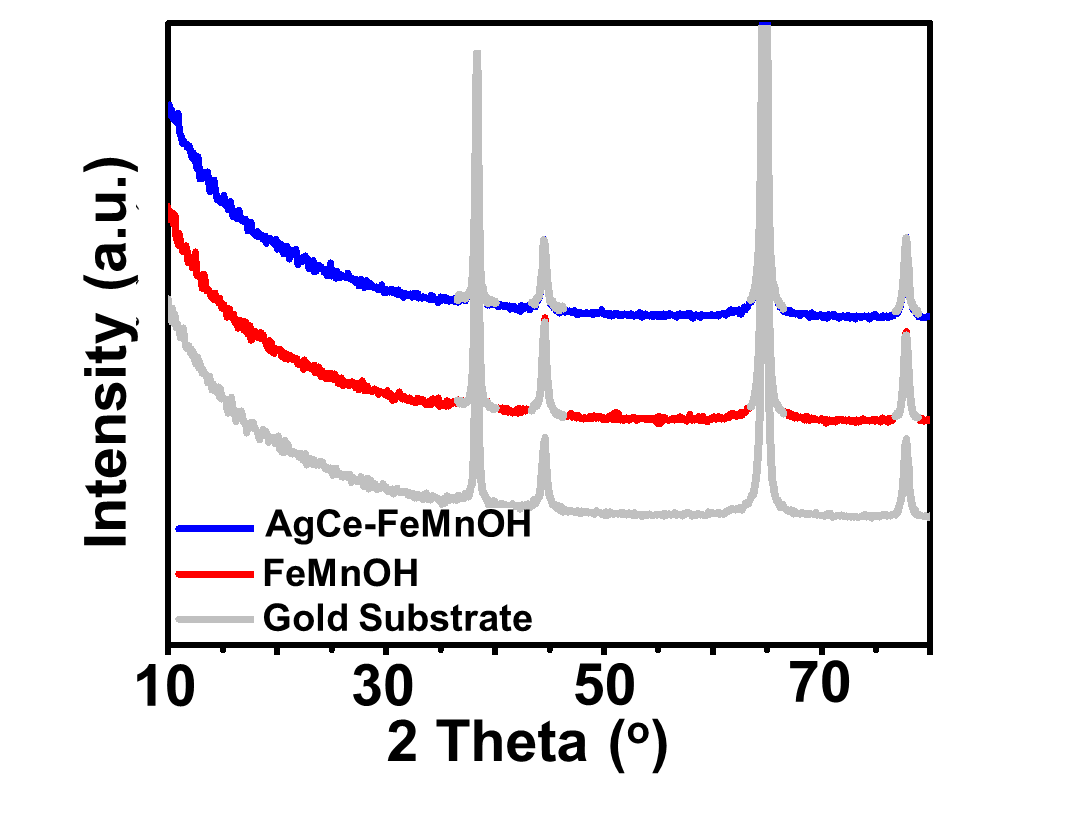


Figure S2. GI-XRD patterns of Au substrate, FeMnOH, and AgCe-FeMnOH catalysts coated in Au substrate, showing amorphous feature of the coated catalyst film.


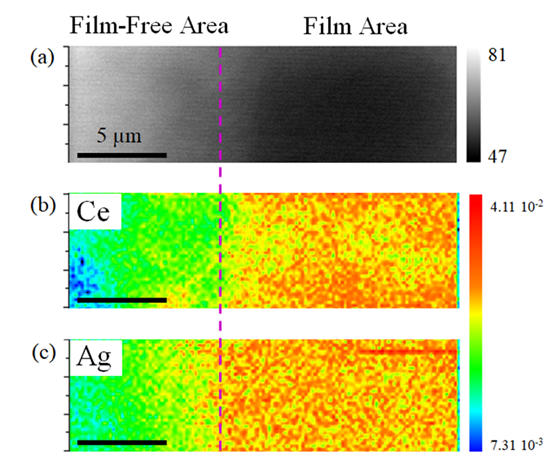


Figure S3. The elemental mapping of Auger electron spectroscopy (AES). (a) The secondary electron images of AgCe-FeMnOH film. The elemental distribution mapping of (b) Cerium and (c) silver corresponding to the area of film coverage shown in (a). The scale bar = 5 µm in both (b) and (c).


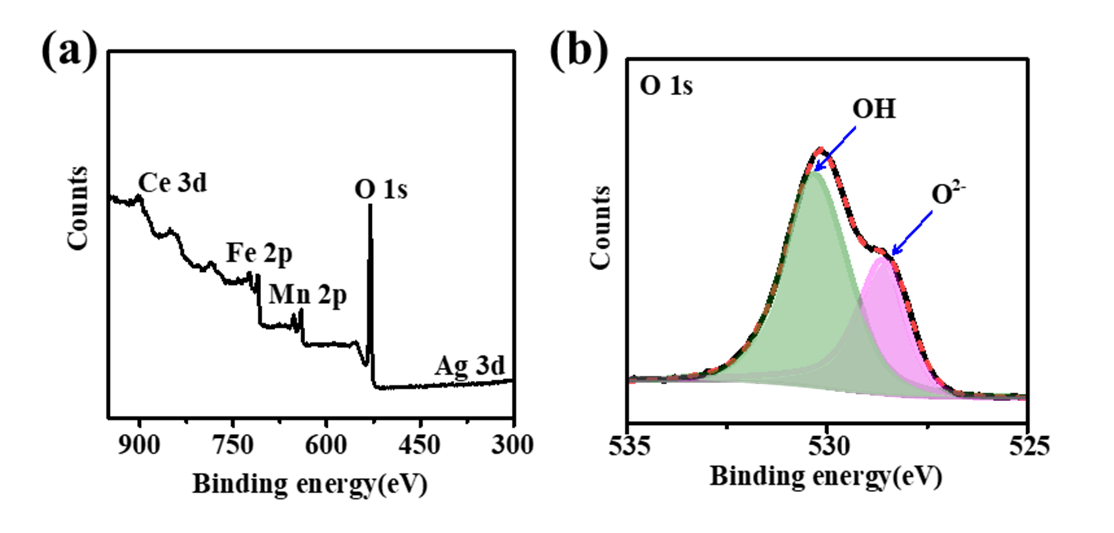


Figure S4. The XPS spectra of AgCe-FeMnOH thin film on a silicon substrate. The survey (a) and O 1s (b) spectra. The O 1s spectra also show two peaks at 528.9 and 530.4 eV, attributed to O^2-^ and OH^-^,^1^ the black line represents the experimental data and the red line corresponds to the fitted result.


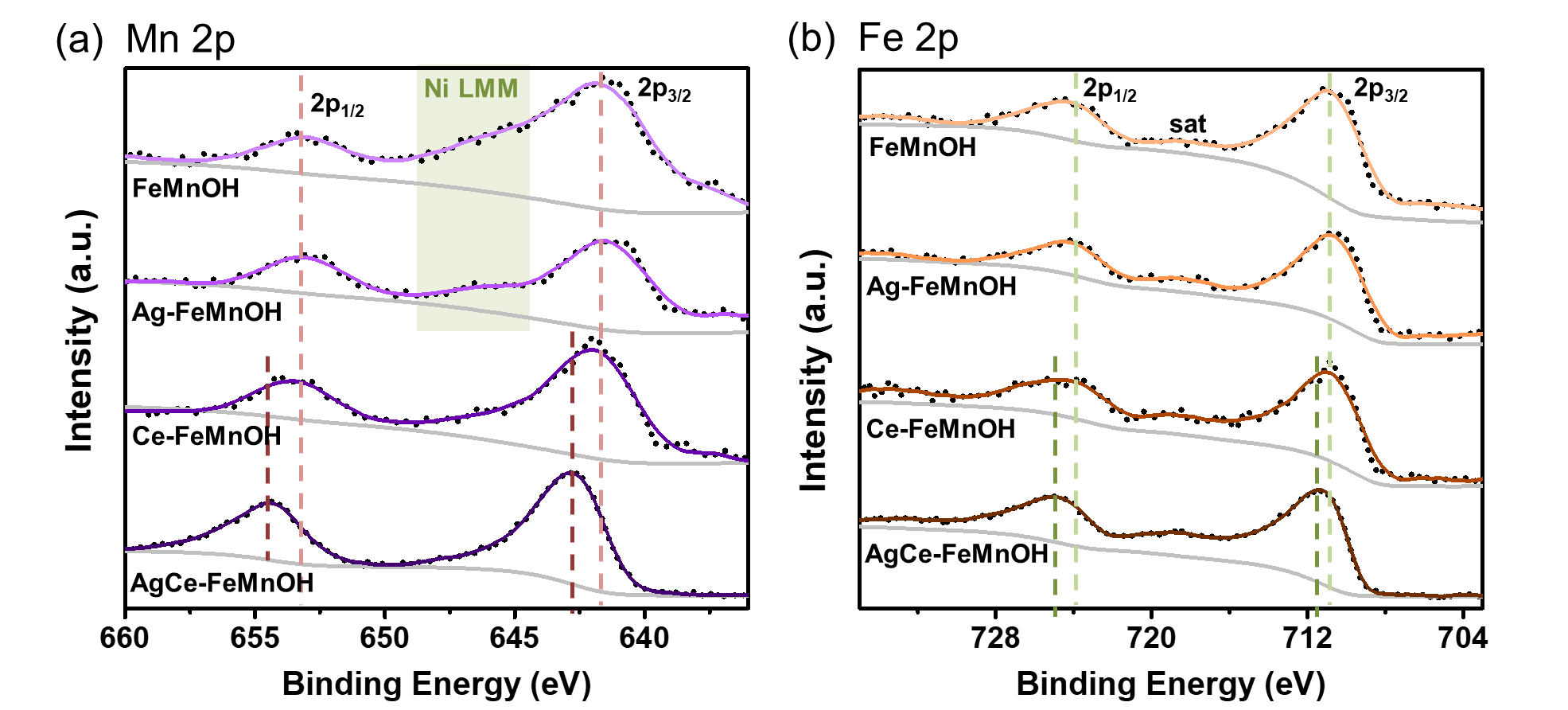


**Figure S5.** High-resolution XPS spectra of (a) Mn 2p and (b) Fe 2p regions for FeMnOH, Ag-FeMnOH, Ce-FeMnOH, and AgCe-FeMnOH. All samples display the characteristic Mn 2p_1/2_, Mn 2p_3/2_, Fe 2p_1/2_, and Fe 2p_3/2_ peaks, with a weak satellite feature in Fe 2p and a Ni LMM Auger contribution from the Ni foam substrate in the Mn 2p region. The dual-doped AgCe-FeMnOH exhibits the most pronounced shift, evidencing synergistic charge redistribution between dopants and the FeMnOH framework and consistent with the enhanced OER activity of the doped catalysts. All spectra shown already referenced by C1s 284.6 eV.


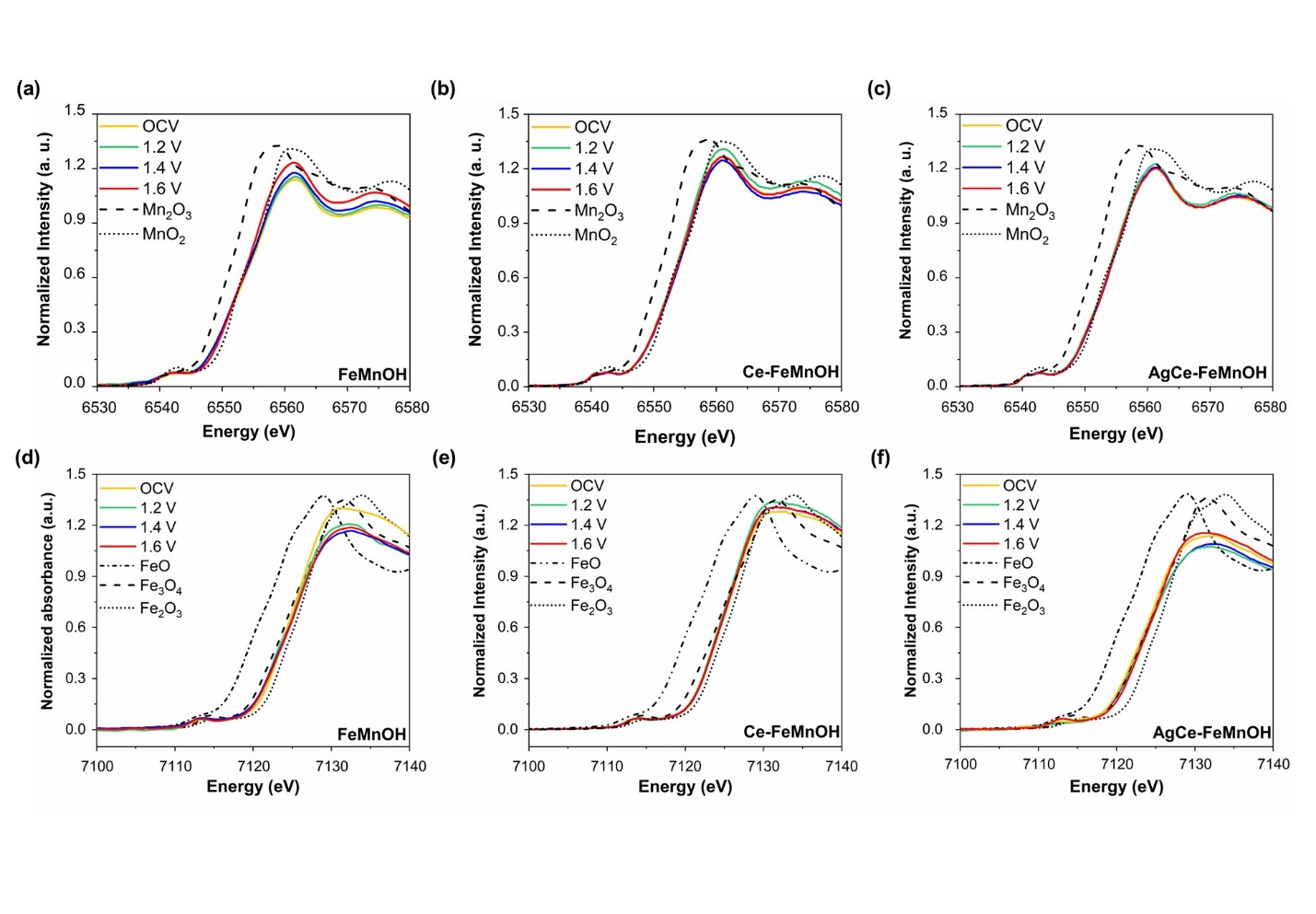


Figure S6**.** Operando XANES analysis of Mn and Fe K-edges under varied OER potentials. Panels (a–c) show Mn K-edge XANES spectra for FeMnOH, Ce-FeMnOH, and AgCe-FeMnOH, respectively, while (d–f) display the corresponding Fe K-edge spectra. All measurements were conducted under salty alkaline conditions (0.1 M KOH + 1 M NaCl).


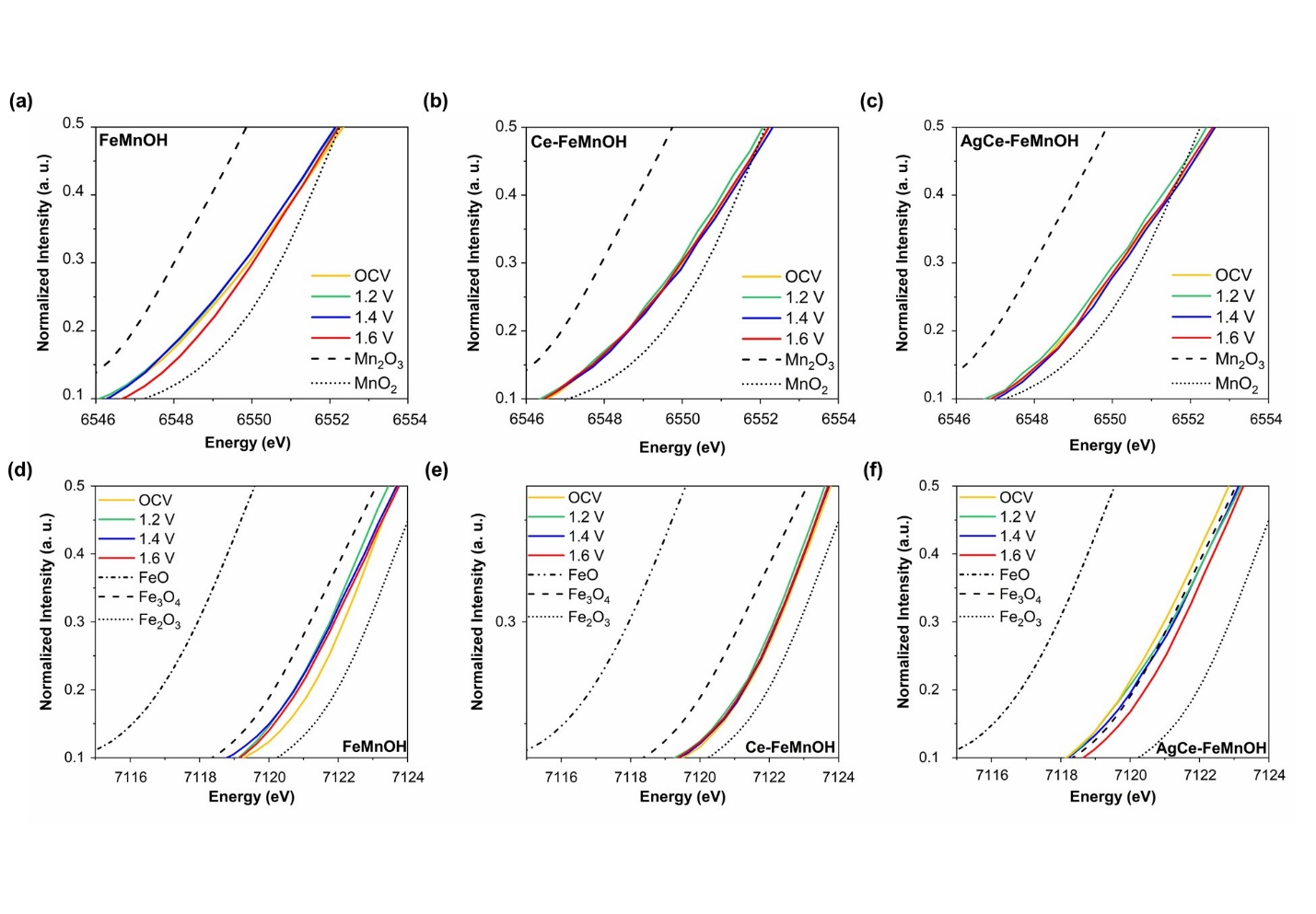


**Figure S7.** Magnified XANES spectra of the Mn and Fe K-edge front peaks extracted from Figure S5. The enlarged views highlight the edge-energy regions, which further used to determine the oxidation state of the metals. Panels (a–c) show Mn K-edge XANES spectra for FeMnOH, Ce-FeMnOH, and AgCe-FeMnOH, respectively, while (d–f) display the corresponding Fe K-edge spectra. All measurements were conducted under salty alkaline conditions (0.1 M KOH + 1 M NaCl).


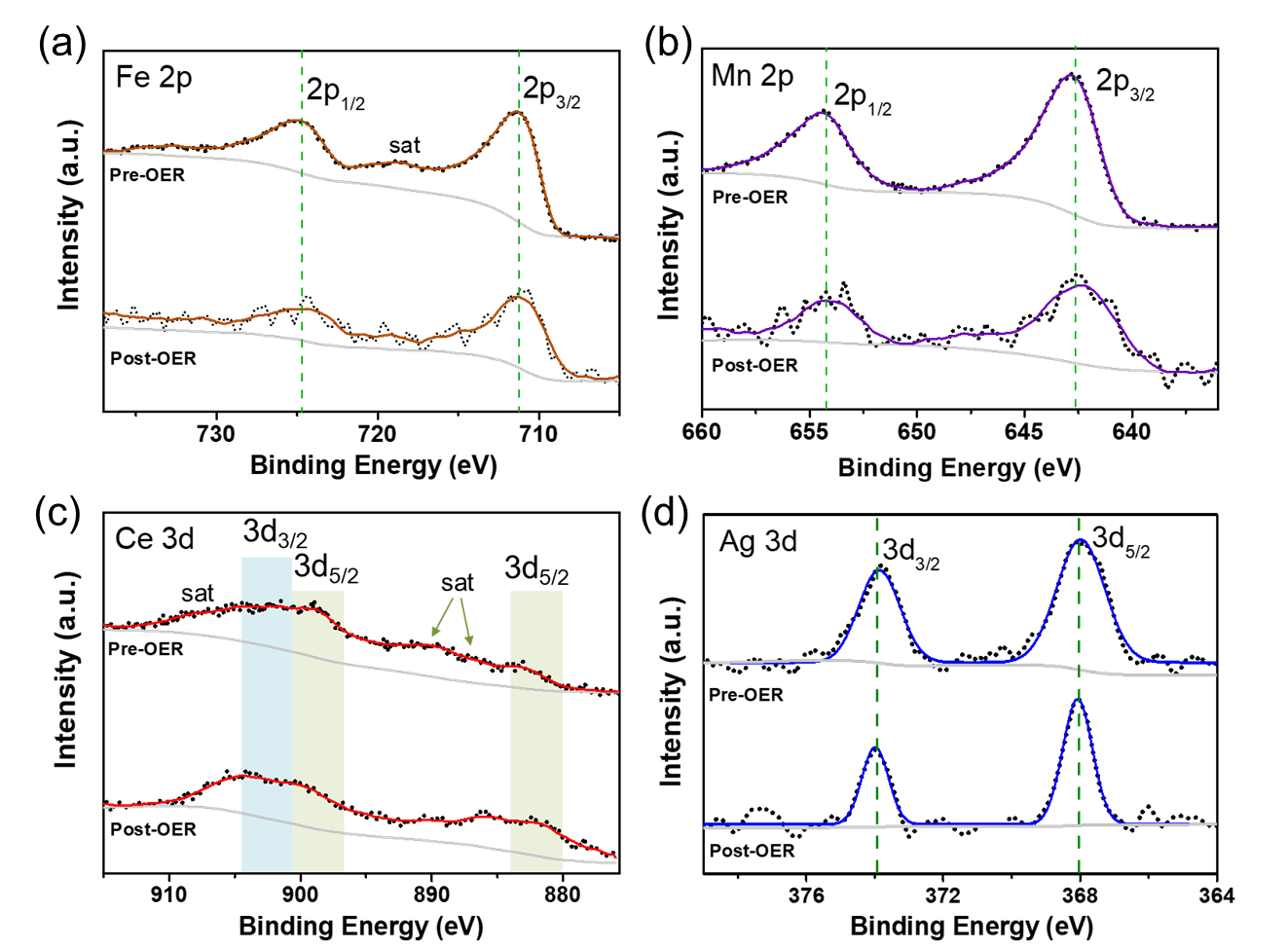


**Figure S8.** Ex-situ XPS spectra of AgCe–FeMnOH thin comparison between fresh sample (pre-OER) and after 5 hours stability under alkaline seawater (post-OER). The test conditions correspond to that demonstrated in Fig. 4. The high-resolution scans of (a) Fe 2p displays only modest variations after OER, indicating slight changes in Fe oxidation while maintaining framework stability. (b) Mn 2p remains essentially unchanged, consistent with stable Mn coordination. The dopants of (c) Ce 3d, and (d) Ag 3d show that cerium retains its mixed Ce^3+^/Ce^4+^ features with only slight shifts, and silver remains metallic (Ag^0^) with no detectable chemical environment change confirming the persistence of its redox states. Experimental data are shown in black dots with fitted curves are overlaid, and background spectra in gray line. All spectra shown already referenced by C1s 284.6 eV.


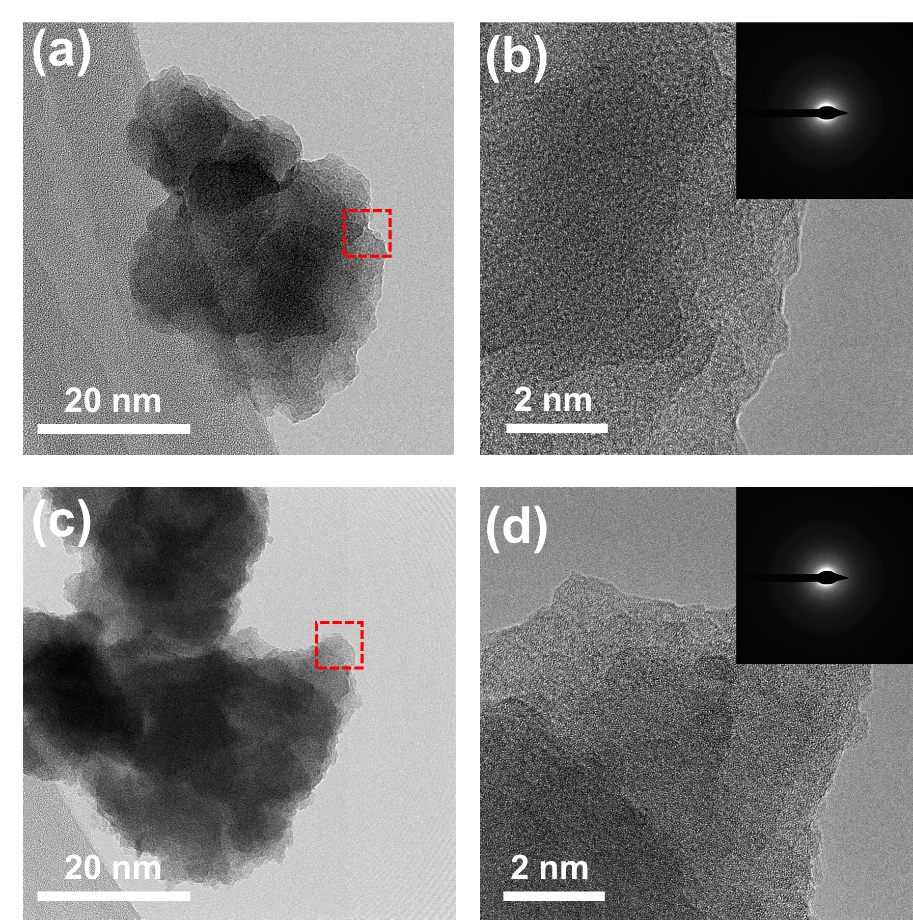


Figure S9. TEM and HRTEM images of AgCe-FeMnOH powder before OER (a,b) and after OER stability for 5 hours in alkaline seawater electrolysis (c, d), the same test conditions as demonstrated in Fig. 4. Insets in (b) and (d) show the corresponding SAED patterns. Both pre- and post-OER samples display a featureless amorphous structure with no lattice fringes or diffraction spots, confirming that the catalyst preserves its amorphous nature and does not undergo structural reconstruction during the operation.

Table S1. ICP-MS analysis of the element composition of the film catalyst deposited on the Ni foam.

| Samples | Molar ratio  in precursor | Molar ratio  in catalyst |
| --- | --- | --- |
| FeMnOH | 3:1  (Fe:Mn) | 2.89:1  (Fe:Mn) |
| Ce-FeMnOH | 0.3:2.7: 1  (Ce:Fe:Mn) | 0.3:2.73:1  (Ce: Fe:Mn) |
| Ag-FeMnOH | 0.09:2.91:1  (Ag:Fe:Mn) | 0.01:2.95:1  (Ag:Fe:Mn) |
| AgCe-FeMnOH | 0.09:0.21:2.7:1  (Ag:Ce:Fe:Mn) | 0.01:0.22:2.96:1  (Ag:Ce: Fe:Mn) |

Table S2. Summary of AEM Electrolyzer Stability and Operating Conditions in Alkaline Seawater Splitting. In this work, AgCe–FeMnOH system maintains long-term stability (250 h) at higher current applied and larger electrode active area.

| OER catalyst | Electrode active area | Current applied (current density) | Stability duration | Reference |
| --- | --- | --- | --- | --- |
| AgCe-FeMnOH | 25 cm^2^ | 5.0 A  (0.2 A/cm^2^) | 250 hours | This work |
| Os-Ni_4_Mo/MoO_2_ | 16 cm^2^ | 4.0 A  (0.2 A/cm^2^) | ≈ 200 hours | *Adv. Mater.* (2024)^4^ |
| RuMoNi | 2 cm^2^ | 1.0 A  (0.5 A/cm^2^) | 240 hours | *Nat. Commun.* (2023)^5^ |
| CrO_4_^2—^NiFe/Cr_2_O_3_/NF | 1 cm^2^ | 0.5 A  (0.5 A/cm^2^) | 200 hours | *Nat. Commun.* (2024)^6^ |
| F-FeNi-LDH | 1 cm^2^ | 0.5 A  (0.5 A/cm^2^) | 80 hours | *Adv. Funct. Mater.* (2025)^7^ |
| NiFeLDH-FLPs | — | (0.2 A/cm^2^) | 250 hours | *Angew. Chem., Int. Ed.* (2025)^8^ |

Table S3. The ICP analysis metal composition of the electrolyte, before, after 24, and after 200 hours stability of AgCe-FeMnOH under 2 M KOH seawater, showing no metal leached out to the electrolyte during the electrolysis. The electrolyte was kept at around 500 mL during the test.

| Electrolyte Samples | Fe (ppm) | Mn (ppm) | Ce (ppm) | Ag(ppm) |
| --- | --- | --- | --- | --- |
| Before electrolysis | ND (<0.01) | ND (<0.01) | ND (<0.05) | ND (<0.01) |
| After 24 hours electrolysis | ND (<0.01) | ND (<0.01) | ND (<0.05) | ND (<0.01) |
| After 200 hours electrolysis | ND (<0.01) | ND (<0.01) | ND (<0.05) | ND (<0.01) |

REFERENCES

1. R.-H. Jhang, C.-Y. Yang, M.-C. Shih, J.-Q. Ho, Y.-T. Tsai, C.-H. Chen, *J. Mater. Chem. A* 2018, **6**, 17915–17928.
2. M.-C. Shih, R.-H. Jhang, Y.-T. Tsai, C.-W. Huang, Y.-J. Hung, M.-Y. Liao, J. Huang, C.-H. Chen, *Small* 2019, **15**, 1903363.
3. T. A. Zegeye, W.-T. Chen, C.-C. Hsu, J. A. A. Valinton, C.-H. Chen, *ACS Energy Lett*. 2022, **7**, 2236–2243.
4. D. Liu, X. Wei, J. Lu, X. Wang, K. Liu, Y. Cai, Y. Qi, L. Wang, H. Ai, Z. Wang, *Adv. Mater.* 2024, **36**, e2408982.
5. X. Kang, F. Yang, Z. Zhang, H. Liu, S. Ge, S. Hu, S. Li, Y. Luo, Q. Yu, Z. Liu, Q. Wang, W. Ren, C. Sun, H.-M. Cheng, B. Liu, *Nat. Commun*. 2023, **14**, 3607.
6. Z. Cai, J. Liang, Z. Li, T. Yan, C. Yang, S. Sun, M. Yue, X. Liu, T. Xie, Y. Wang, T. Li, Y. Luo, D. Zheng, Q. Liu, J. Zhao, X. Sun, B. Tang, *Nat. Commun*. 2024, **15**, 6624.
7. J. Mu, C. Yu, X. Song, L. Chen, J. Zhao, J. Qiu, *Adv. Funct. Mater*. 2025, **35**, 2423965.
8. J. Zhu, T. Cui, J. Chi, T. Wang, L. Guo, X. Liu, Z. Wu, J. Lai, L. Wang, *Angew. Chem. Int. Ed.* 2025, **64**, e202414721.
